# Embarrassing Product, Image Type, and Personal Pronoun: The Mediating Effect of Body Imagery

**DOI:** 10.3389/fpsyg.2021.796998

**Published:** 2022-01-19

**Authors:** Shenghong Ye, Haoyun Yan, Zhengyu Lin, Zan Huang

**Affiliations:** School of Management, Jinan University, Guangzhou, China

**Keywords:** embarrassment, body imagery, packaging image, dual-process model, person pronoun, purchase intention

## Abstract

Consumers often feel embarrassed when buying products like condoms, hemorrhoid cream, and beriberi cream in crowded pharmacies. There is an interesting phenomenon in life: Some beriberi creams use the images of a “real foot”, while others use the images of a “cartoon foot.” Imagine if a young woman needed to go to a retail store for beriberi cream that would embarrass her, she would choose a “real foot image” or a “cartoon foot image” beriberi cream? It has been shown that the embarrassment of these products has a strong negative impact on consumer buying behavior. Previous researches have explored how changing packaging elements of embarrassing products (e.g., color/design/image placement) can effectively reduce consumer embarrassment. However, few have examined the impact of different image types of embarrassing product packaging (artificial vs. natural) with embarrassment. Therefore, this research explores the effect of image types (artificial vs. natural) on consumers’ willingness to purchase embarrassing products and reveals the mechanisms of the underlying effects. The results show that natural images can lead to lower purchase intention of embarrassing products when the advertisement uses first-person pronouns due to the mediating role played by negative body imagery. However, there is no significant difference in purchase intention between different image types in the third-person pronouns. Finally, this paper discusses its contributions and limitations.

## Introduction

People always encounter some embarrassing products in life. In fact, academics have found consumers experiencing embarrassment when buying “embarrassing products” like condoms (Dahl et al., 2001), women’s care lotion (Lau-Gesk and Drolet, 2008), and so on. When the consumer is embarrassed, they will reduce willingness to buy embarrassing products (Penz and Stoettinger, 2004; Wang et al., 2013) and willingness to buy (White and Dahl, 2007; Berger and Ward, 2010), and lower evaluation of embarrassment-induced advertising (Puntoni et al., 2015), and some consumers even spread negative word of mouth of the product (Wu and Mattila, 2013). Clearly, paying attention to consumer embarrassment can help enterprises to retain loyal customers and improve product sales and enterprise profits.

For embarrassing products, product packaging elements are an essential factor affecting consumer purchase behavior. Because the vast majority of consumers are reluctant to buy embarrassing products in retail stores, they will try to “hide their emotions” and hide their embarrassment (Isabella et al., 2015). To get rid of negative emotions, consumers end purchases quickly and flee stores (Londono et al., 2017). Enlightened thinking refers to thinking whereby consumers can find solutions to problems in a short time based on limited knowledge. To minimize the time of choice, consumers will use shortcuts in decision making and use enlightened thinking (Laksmidewi et al., 2017). Product packaging elements can provide a shortcut as “external clues” and will encourage consumers to think in a more enlightened way. Therefore, packaging elements are important for embarrassing products.

Among the many product packaging elements, the images on the packaging are the most consumer-appealing part. The packaging of embarrassing products often contains images associated with body parts: for example, foot odor ointment will be printed with a foot, and anti-hair loss products printed with a bald man. In the packaging design of embarrassing products, there are often two types of these images: natural images and artificial images. So, which image type can embarrassing products use to make a better impact? There is no definite conclusion at present. There are conflicting conclusions on image types (natural vs. artificial). Studies have proposed that using natural images can enhance consumers’ expectations (Kim et al., 2019). However, some scholars believe that artificial images can enhance expectations for specific product properties (Cai and Chi, 2021). These two contrasting findings exist, most likely because the researchers did not distinguish between the categories of products. Due to the particularity of embarrassment and the high relevance of the image content to the body, the potential mechanism for the influence of image type (natural vs. artificial) on the willingness to buy an embarrassing product may be very different. However, no relevant research has been found in the field of embarrassing research. To this end, this study takes the image type of embarrassing product (artificial vs. natural) as the breakthrough point, based on emotion-cognitive double processing theory and body imagery, to explore the impact of different image types on consumer willingness to buy and the psychological mechanism and possible boundary conditions therein.

The rest of this article is organized as follows: Section “Literature Review and Hypotheses Development” reviews the literature and proposes the hypotheses, Sections “Pretest” and “Study 1: The Effect of Image Type on the Willingness to Buy Embarrassing Products” test the effect of image types (artificial vs. natural) on consumers’ willingness to purchase embarrassing products. Section “Study 2: The Mediating Role of Body Imagery” examines the robustness of the effect as well as the underlying mechanism. Section “Study 3: The Moderating Role of Personal Pronouns” explores the possible boundary conditions therein of the effect. Section “Conclusion and Discussion” concludes and discusses the article.

## Literature Review and Hypotheses Development

### Consumer Embarrassment and Embarrassment Products

Embarrassment is a negative emotion when individuals violate social rules shared by the group (Goffman, 1963). Studies on embarrassment in other areas focus more on social situations and interpersonal situations, while research in the marketing community focuses on consumer embarrassment. Consumer embarrassment refers to an unpleasant and distressing emotional state occurring mainly because the consumer thought (realistically or imagined) that bystanders would negatively view or evaluate embarrassing events occurring by the consumer (Dahl et al., 2001).

Embarrassing products are very closely related to consumer embarrassment. They are products that can easily cause consumer embarrassment are embarrassing products, such as personal hygiene supplies (Katsanis, 1994), adult magazines (Berkowitz, 2006), and so on. There is a recent study, which surveyed the AOL Consumer group, aggregated into 173 embarrassing products, and classified them into 19 product types (Jones et al., 2018). Through the analysis of these 19 product types is available, we found that embarrassing products have two main characteristics. The first is that consumers may risk being stigmatized when buying embarrassing products; and the second characteristic is the innovation point of this article, that is, embarrassing products are generally highly related to the body, such as hair growth agent being related to consumer body head hair and hemorrhoid paste and medical ointment being related to the sick parts of the consumer’s body. The second feature is the innovation point of this study. The previous studies on embarrassing products have ignored this feature of embarrassing products. Current research on embarrassing products focuses on their impact on consumer behavior and better marketing strategies (such as changing the purpose of purchase and the way products are displayed). Studies have found that when it comes to buying awkward products, consumers will reduce embarrassment by going to less crowded stores, waiting for other customers to leave the aisle, not asking for help from employees, and adding more items to their baskets to distract others (Blair and Roese, 2013). As a result, many studies have explored what factors affect the impact of embarrassing products on consumers. In terms of buying purposes, consumers experience significantly less embarrassment when they buy Viagra (an impotence drug developed by Pfizer) to increase pleasure, rather than to treat dysfunction (Krishna et al., 2015). As for the way of product display, studies have found that when non-embarrassing products (such as paper towels and lotion) purchased together are complementary or related to embarrassing products (such as condoms), consumers’ embarrassing experience will increase (Nichols et al., 2015). But no research yet discusses the impact of packaging images of embarrassing products on consumers.

In conclusion, consumer embarrassment is an independent self-conscious mood, and this self-conscious sentiment is negative. Embarrassment gives consumers a certain degree of discomfort and differences from other self-conscious emotions. Furthermore, the embarrassing products associated with them are stigmatized and highly relevant to the physical domain. There is currently a lack of research on the packaging images of embarrassing products. Therefore, this paper attempts to fill this gap by extending and enriching relevant studies.

### Image Types: Natural vs. Artificial

Packaging images can be divided into natural and artificial images. Natural pictures are more on-site and authentic, such as photos. Artificial pictures are more inauthentic and idealistic, such as non-realistic pictures. A study by Smith et al. (2015) selects 12 kinds of food in the market and created two types of images for each of them: one is a photo, the other is an artist’s painting. The audience often considers photos to be more evidential or documentary. In addition, it has been shown that the visual effects of unprocessed foods symbolize naturality. Facing the same juice, consumers have a more natural perception of unprocessed images than processed images (Machiels et al., 2016). Overall, natural images are more specific, while artificial images are more abstract. Because symbolic artificial images contain more metaphorical significance (Velasco and Spence, 2019) than natural images directly displaying the product itself, abstract artificial images contain more complex information. Natural images will contain information more simple and intuitive, and people will be more familiar with natural images, so specific natural images are better identifiable.

Based on the emotion-cognition dual-process model, when people receive different information, cognitive or emotional processing systems will be initiated based on their information characteristics (Zhao et al., 2011). When the individual faces the more complex the information, the more connotation, the less intuitive it is, the more cognitive resources are allocated when processing information (McQuarrie and Mick, 1999), then the initiating cognitive system will be activated (Kim et al., 2012). The cognitive processing system processes complex information, the process is complex and slow; When individuals face with intuitive and emotional information, initiating emotional processing methods (Aaker et al., 1986). The emotional processing system performs intuitive, automated emotional response (Epstein, 1994), with simple and fast processes.

Artificial images contain more abstract and complex information than natural images, so processing such images requires more cognitive effort (Gil-Pérez et al., 2019), and thus artificial images prioritize the cognitive system. Since natural images are more intuitive and vivid, and the vividness of images inspires more consumer levels of emotion (Shiv and Nowlis, 2004), natural images preferentially initiate emotional systems. Two different processing modes, cognitive and emotional, have different effects on consumer attitudes, evaluation, and decision making (Zauberman et al., 2006). The priority of the cognitive system by artificial images will enable consumers to see more embarrassing products rationally and pay more attention to the economic value and functional utility of embarrassing products, thus promoting the willingness to buy. The priority of the emotional system will cause more embarrassment and will negatively affect consumers’ willingness to buy. Studies have shown that embarrassing emotions will reduce the willingness to buy and repurchase embarrassing products (Berger and Ward, 2010), so the embarrassing products packaged in natural image products will lead to lower consumers’ willingness to buy compared to artificial images. Taken together, we propose the following hypothesis:

*Hypothesis* 1: The natural perception of images is negatively related to embarrassing products’ purchase intention.

### The Mediating Role of Body Imagery

From the literature review described above, embarrassment is defined as a negative self-conscious emotion (Pila et al., 2016). This mood of negative self-awareness is highly correlated to the physical domain, such as embarrassment occurs when an individual falls, is physically uncontrolled, or accidentally exposed or displayed (Slater and Tiggemann, 2011), and body-related embarrassing experiences may be particularly prominent during adolescence and early adulthood (Harter, 2015). We studied the commonality of embarrassing products and found that many embarrassing products are related to the consumer’s body. Therefore, we used the variable of body imagery.

Body imagery is a concept composed of a multi-dimensional composition of perception, influence, and cognition associated with the body. Body imagery is a fundamental component of the self-concept (Cash, 2004). Based on information processing theory, consumers will regard body imagery as a cognitive bias, and individuals will form self-schema about body and diet in their minds according to their previous experiences. When the negative stimulus associated with this schema is received, individuals are stimulated to produce negative body imagery due to the presence of a “selective interpretation bias” (Vahia, 2013). This negative evaluation of self-body imagery leads individuals to negative emotions such as individuals feeling depressed and is more likely to cause various psychotic problems such as depression, suicide, and social isolation (Angelakis et al., 2016).

Natural images are a more realistic, concrete image and the priority evokes the emotional system. When consumers see natural images on embarrassing products, they are more likely to associate with their defective body parts and feel more negative body imagery. For example, frequent use of highly visual social media (such as Instagram and Snapchat) relative to low-visual social media (such as Facebook) leads to adolescent body imagery concerns, which leads to poor mental health conditions (Marengo et al., 2018). The negative body imagery is generally associated with negative outcomes, therefore, strong negative body imagery may have negative effects on consumers’ desire to buy. Artificial images are more abstract and give priority to starting the cognitive system. The negative body imagery that consumers feel is not so strong, which will cause them to look at these schema-related negative stimuli more rationally and pay more attention to the economic value and functional utility of embarrassing products, and so consumers’ willingness to purchase will not be weakened. Taken together, we propose the following hypothesis:

*Hypothesis* 2: Body imagery mediates the relationship between image type and embarrassing products’ purchase intention.

### The Moderating Role of Personal Pronouns

Personal pronouns refer to the alleged mark of all participants in that event in a verbal event. First-person pronouns are reference marks of the speaker in a speech event, and third-person pronouns indicator refers to the others except for the speaker himself and the person they talk to Brener (1983). Relatively speaking, a first-person narrative is more personal and subjective, and a third-person narrative is more objective. There is some research on personal pronouns in the field of marketing. For example, studies between personal pronouns and consumer-brand relationships indicate that consumers respond differently to language that implies relationships, while different personal pronouns can imply different relationships (Sela et al., 2012). In addition, studies explore the application of personal pronouns in brand naming. Studies have been shown that “I” and “My” influence consumer preferences and psychological mechanisms under different conditions, and the use of “My” can enable subjects to have subjective ownership (Kachersky and Palermo, 2013). Furthermore, studies have shown that “I” represents a concern for oneself, while “You” is a concern for oneself and others (Pennebaker, 2011). Existing research on personal pronouns mainly discusses the application of singular and plural first-person pronouns and first-person pronouns and second-person pronouns in advertisements (Sela et al., 2012). Although third-person pronouns are widely used in advertising and narrative, there is no research on the application of third-person pronouns in advertising and the difference between first-person pronouns and third-person pronouns in the field of marketing. Therefore, this study will explore the application effect of first-person pronouns and third-person pronouns in embarrassing product advertising slogans.

The level of personal relevance refers to the extent to which personal relevance is triggered by some external stimulus, prompting an individual to become more self-absorbed in dealing with things. Research has shown that personal relevance occurs when people process information about themselves or their own experiences (Burnkrant and Unnava, 1995). Personal pronouns are the most common means of manipulating the level of personal relevance. Some studies have suggested that the second-person pronoun “you” and the first-person pronoun “I” can make consumers feel more self-relevant from brand names or advertising than the third-person pronoun (Fennis, 2012). As a result, the adoption of first-person pronouns in advertising gives consumers more perceived personal relevance than third-person pronouns and enables consumers to think that information is aimed at the consumer himself. When consumers receive these schema-related negative stimuli, they feel more negative body imagery, thus triggering a higher level of embarrassment and reducing their willingness to buy embarrassing products. On the contrary, when using third-person pronouns in advertisements, consumers perceive that the level of personal relevance will be low. Their reminiscent of the psychological simulation will more related to others, not their own defective body so that the effect of the image types of embarrassing product packaging on consumers’ willingness to buy will disappear. Therefore, the following hypothesis is proposed:

*Hypothesis* 3: Personal pronouns moderate the relationship between body imagery and embarrassing products’ purchase intention such that the relationship is stronger when the advertisement uses first-person pronouns.

## Pretest

Before the formal experiment, the appropriate packaging images (natural vs. artificial) were selected as the experimental materials through the pretest. The selection of experimental materials was based on previous research, which surveyed 60 participants and asked them to list “the three products most embarrassing when shopping.” The final survey was summarized with 173 embarrassing products and finally classified into 19 product categories (Jones et al., 2018). Considering that images of certain product categories involve personal privacy and are inconvenient to display, the three categories of hemorrhoid cream, hair restorer, and medical ointments were selected as experimental materials as they ranked 3rd, 5th, and 7th in the 19 product categories, all above average. All three products have been established as embarrassing products in previous literature (Wang et al., 2013; Jones et al., 2018).

This paper selected artificial and natural images for these three product categories. This paper makes natural images and artificial images respectively for these three categories. We mix the images in the experiment. Then, we informed participants of the meaning and characteristics of artificial and natural images and require them to classify the experimental material into artificial and natural images. The data showed that most participants could successfully classify (*p*’s < 0.05), indicating successful manipulation. In the following formal experiments, hemorrhoid cream, beriberi cream (medical ointment), and hair restorer were used as the experimental materials.

## Study 1: The Effect of Image Type on the Willingness to Buy Embarrassing Products

Study 1 examined the different effects of two image types (artificial vs. natural) on willingness to buy on different product categories (embarrassing products vs. neutral products).

### Experimental Design and Subjects

A between-subject design of 2 (packaging images: natural vs. artificial) × 2 (product categories: embarrassing products vs. neutral products) was used in Study 1. We recruited 160 students from a large university in South China, and the participants were randomly assigned to one of the four groups^1^^–^^4^. A total of 147 valid questionnaires were received (male 49%, female 51%).

### Experimental Procedures

First, participants were told they needed to buy some items (hemorrhoid cream/vitamin C), and the experiment introduced the use of hemorrhoid cream/vitamin C, randomly displaying one of four images (artificial image of hemorrhoid cream/natural image of hemorrhoid cream/artificial image of vitamin C/natural image of vitamin C) to ensure they could fully understand the products purchased.

Second, participants were required to rate packaging images on artificial and natural scales as manipulation (1 = natural, 7 = artificial; α = 0.846). Then participants needed to rate the level of embarrassment they experienced (three items: not embarrassed at all/very embarrassed, not uncomfortable at all/very uncomfortable, and not awkward at all/very awkward, α = 0.930) (Dahl et al., 2001) and the willingness to buy embarrassing products (three items: this incites me to buy the product/it is likely that I will spend money on this product/I want to have this product, α = 0.911) (Puntoni et al., 2015). The above-mentioned measurement scales are all 7-point scales and have been adjusted and adapted to the situation of this experiment.

Finally, participants were thanked for their participation and debriefed after completing the questionnaire.

### Results

#### Manipulation Check

The first is to test the manipulation effect of packaging image type. A *t*-test result showed that the artificial perception of artificial images was significantly higher than that of natural images both in hemorrhoid cream (*M*_artificial_ = 5.29 vs. *M*_natural_ = 3.91; *t* = 4.221, *p* < 0.001) and vitamin C (*M*_artificial_ = 5.26 vs. *M*_natural_ = 1.45; *t* = 4.221, *p* < 0.001), indicating that the manipulation of image type is successful. Then we conducted a *t*-test on the manipulation of product categories, and the results showed that the level of embarrassment for the hemorrhoid cream group was significantly higher than that of the vitamin C group (*M*_hemorrhoid cream_ = 5.06 vs. *M*_vitamin C_ = 2.26; *t* = −15.810, *p* < 0.001), indicating that the manipulation of product categories is successful.

#### Hypothesis Test

**FIGURE 1:**
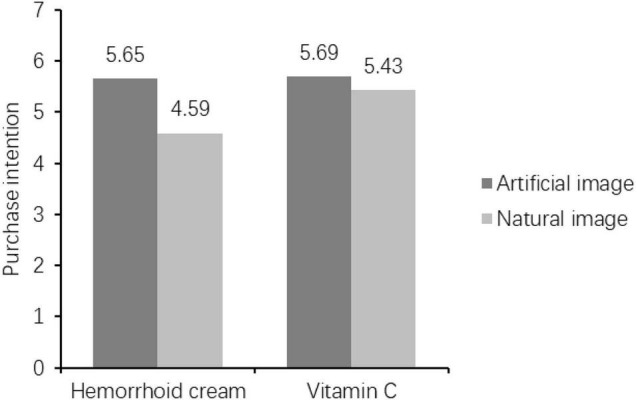
The purchase intentions of different situations.

To prove different purchase intentions under different levels of embarrassment, this study performed one-way ANOVA for different product categories. In the hemorrhoid cream condition, participants were more likely to buy embarrassing products with artificial images (*M*_artificial_ = 5.65 vs. *M*_natural_ = 4.59; *F* = 34.094, *p* < 0.001, see Figure 1). However, there is no significant difference in a willingness to buy embarrassing products for the two image types in the vitamin C condition (*F* = 1.068, *p* = 0.305). These results suggest that, compared to artificial images, natural images can lead to a lower willingness to buy embarrassing products. Therefore, H1 was supported in Study 1.

### Discussion

Study 1 investigated the effect of packaging image types on purchase intention of embarrassing products, and successfully verified H1. However, Study 1 still has some deficiencies, such as that the experimentally simulated scenarios lack authenticity. Furthermore, the potential mechanism of effect has not been explored in Study 1, but these will be discussed in Study 2.

## Study 2: The Mediating Role of Body Imagery

The main purpose of Study 2 was to test the mediating role of body imagery in ways of providing an explanation mechanism for the results of Study 1. Meanwhile, Study 2 adopted a new embarrassing product category (beriberi cream) that was different from Study 1, and increased the realism of the experimental scenarios to enhance the robustness of the experimental results.

### Experimental Design and Subjects

We recruited 120 students from a large university in South China, and the participants were randomly assigned to either an artificial image or a natural image. A total of 103 valid questionnaires were received (male 42.71%, female 57.28%).

### Experimental Procedures

First, participants were told they needed to buy a beriberi cream, and the experiment introduced the use of beriberi cream to ensure they could fully understand the products purchased. Second, participants were given a photo of a shopping scenario at the pharmacy, which had two physicians and another consumer to increase the participants’ realism^5^. Participants were asked to imagine themselves in such a shopping scenario, looking around the entire pharmacy from a first-person perspective. Participants were shown shelf photos with beriberi cream and randomly saw the product that the package was artificial/natural images^6^.

Next, participants were asked to recall relevant content from the picture they had just seen to test their attention. Subsequently, participants were required to rate packaging images on artificial and natural scales, the willingness to buy embarrassing products, and the level of negative body imagery (six items: I am dissatisfied with my body/I believe that I have a defect in my body/Because

of my body, I avoid appearing in public situations/When I have physical contact with others, I change my movements or body posture to hide the defect/I am often upset when concentrating on my body, α = 0.941) (Puntoni et al., 2015). The above-mentioned measurement scales are all 7-point scales and have been adjusted and adapted to the situation of this experiment. Finally, participants with incorrect perceptions or missed reading checks were not used in analyses.

### Results

#### Manipulation Check

A *t*-test result showed that the artificial perception of artificial images was significantly higher than that of natural images in beriberi cream (*M*_artificial_ = 5.32 vs. *M*_natural_ = 3.92; *t* = 4.615, *p* < 0.001), indicating that the manipulation of image type was successful.

#### Hypothesis Test of the Main Effect

**FIGURE 2:**
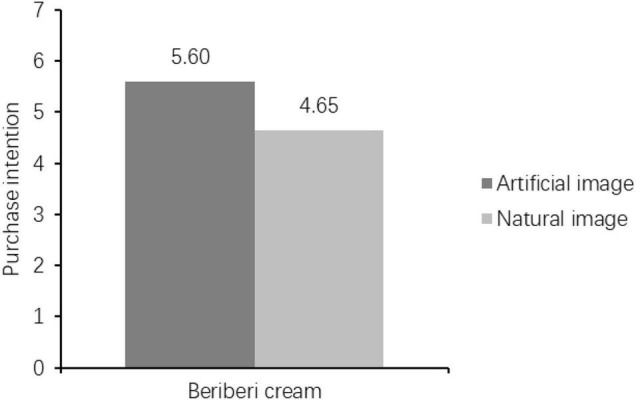
The purchase intentions of different situations.

An ANOVA with the packaging image type as independent variables and embarrassing products’ purchase intention as the dependent variable revealed a significant effect (*M*_artificial_ = 5.3 vs. *M*_natural_ = 4.6; *F* = 8.956, *p* < 0.005, see Figure 2). Therefore, H1 was supported again in Study 2.

#### The Mediation Analysis of Body Imagery

We examined the role of body imagery in the effect of packaging image type on embarrassing products’ purchase intention. Firstly, a mediation analysis with 5,000 bootstrap samples (model 4) with packaging image type as the independent variable, body imagery as the mediator, and purchase intention of embarrassing products as the dependent variable was conducted to test H2. The result revealed a significant mediating effect of body imagery (β = −0.463, SE = 0.065, 95%CI [−0.759, −1.766]), indicating that H2 was supported.

### Discussion

Study 2 modified the stimulus material and increased the realism of the experimental scenarios to increase the validity and applicability of experimental findings. The effect of packaging image type on embarrassing products’ purchase intention was demonstrated again in Study 2. Moreover, Study 2 further investigated the underlying mechanism of the effect. However, Study 2 did not examine the boundary conditions for this effect. In this regard, Study 3 introduced the personal pronoun (first-person pronouns vs. third-person pronouns) as a moderator variable that explores this issue.

## Study 3: The Moderating Role of Personal Pronouns

The aim of Study 3 was to explore the boundary conditions under which embarrassing product image types affect consumer willingness to buy, verifying the moderating role of personal pronouns (first-person pronouns vs. third-person pronouns) in the effect of image types (natural vs. artificial) on embarrassing products’ purchase intention (H3).

### Experimental Design and Subjects

A between-subject design of 2 (packaging images: natural vs. artificial) x 2 (personal pronouns: first-person pronouns vs. third-person pronouns) was used in Study 3. We recruited 240 students from a large South university in China, and the participants were randomly assigned to one of the four groups. A total of 222 valid questionnaires were received (male 49.54%, female 50.45%).

### Experimental Procedures

First, participants were told they needed to buy a hair restorer, then participants were given a photo of a shopping scenario at the pharmacy, which same as Study 2. Participants were asked to imagine themselves in such a shopping scenario, looking around the entire pharmacy from a first-person perspective. Next, participants were randomly shown one of four images^7^^–^^9^. Finally, participants were required to rate packaging images on artificial and natural scales, the willingness to buy embarrassing products, and the level of negative body imagery.

### Results

#### Manipulation Check

A *t*-test result showed that the artificial perception of artificial images was significantly higher than that of natural images in hair restorer (*M*_artificial_ = 5.23 vs. *M*_natural_ = 3.92; *t* = 4.292, *p* < 0.001), indicating that the manipulation of image type is successful.

#### Hypothesis Test of the Mediated-Moderation Effect

First, an ANOVA with the packaging image type as independent variables and embarrassing products’ purchase intention as the dependent variable revealed a significant effect (*M*_artificial_ = 5.3 vs. *M*_natural_ = 4.6; *F* = 4.202, *p* = 0.042). Therefore, H1 was supported again in Study 3.

Second, we took the image type, the interaction between image type and personal pronoun as the independent variable, and body imagery as the dependent variable to conduct an ANOVA. The results showed that image type (*F* = 4.755, *p* = 0.030) and the interaction between image type and personal pronoun (*F* = 16.61, *p* = 0.000) had a significant influence on body imagery. When the first-person pronoun was used, the main effect of image type on body imagery was enhanced (*M*_artificial_ = 5.60; *M*_natural_ = 4.65, *F* = 16.61, *p* = 0.000). When the third-person pronoun was used, the main effect of image type on body imagery was no longer significant (*M*_*artificial*_ = 5.38; *M*_*natural*_ = 5.10, *F* = 1.37, *p* = 0.243).

Thirdly, we conducted regression analysis with body imagery as the independent variable and purchase intention as the dependent variable. The results showed that body imagery had a significant negative effect on purchase intention (β = −0.621, *t* = 3.702, *p* = 0.044).

**FIGURE 3:**
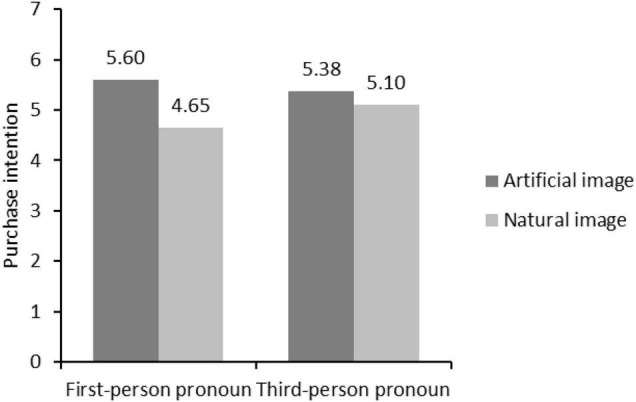
The purchase intentions of different situations.

Finally, in order to test the mediated-moderation effect, we conducted a moderated mediation analysis with image type as the independent variable, body imagery as the mediator, personal pronouns as the moderator, and purchase intention as the dependent variable (model 7 in PROCESS with 5,000 bootstrap samples and 95% bias-corrected intervals). The results showed that image type (β = 1.875, *t* = 10.956, *p* = 0.000) and the interaction between image type and personal pronoun (β = 0.765, *t* = 8.956, *p* = 0.003) had a significant influence on body imagery, and body imagery had a negative influence on purchase intention (β = −0.774, *t* = 4.202, *p* = 0.042). In addition, a significant index of moderated mediation is revealed (β = −0.876, 95% CI = [−1.212, −0.823], see Figure 3). When the first-person pronoun was used, the mediating effect of body imagery was significant (β = −0.945, 95%CI = [−1.322, −0.756]). When the third-person pronoun was used, the indirect effect of body imagery was no longer significant. Therefore, H3 was supported in Study 3.

### Discussion

In Study 3, the stimulus materials were modified to improve the validity and applicability of the experimental results. Study 3 again verified the effect of packaging image type on purchase intention of embarrassing products. Furthermore, Study 3 demonstrated that the personal pronoun plays a moderating role in the effect of image types of packaging on the willingness to purchase embarrassing products. The interaction of the personal pronoun shows these packaging differences act differently depending on the advertisement in which the personal pronoun.

## Conclusion and Discussion

### Conclusion

The current research advances our understanding of how packaging image type (natural vs. artificial) and personal pronouns (first-person pronouns vs. third-person pronouns) affect consumers’ purchase intention of embarrassing products. Across three experiments, we found that there was an effect of packaging image type on embarrassing products’ purchase intention, repeated the effect found in Study 1 under different experimental contexts, and explored the potential mechanism of the matching effect by examining the mediating roles of body imagery. Furthermore, we prove that this effect was moderated by personal pronouns. To be specific, when advertising is in the first-person pronouns, consumers are more likely to buy embarrassing products with artificial images instead of natural images. When using the third-person pronouns, there is no significant difference in a willingness to buy embarrassing products for the two image types.

### Theoretical Implication

The current research provides several theoretical implications. Firstly, this paper extends the research of mental imagery (body imagery) for the field of marketing. Body imagery is defined as the individual’s depiction of their body in his mind, which belongs to a kind of mental imagery. The previous research on body imagery mostly from the aspects of sociology and psychology, and few studies from the aspects of marketing. This study expands individual body imagery and explores its impact on consumer purchasing decisions. Through empirical studies, this study demonstrates that negative body imagery leads to reduced consumers’ willingness to buy embarrassing products. Furthermore, this study reveals the interplay between mental imagery (body imagery) and emotions (embarrassment) in the context of decision making.

Secondly, this research enriches the literature in the field of embarrassment and embarrassing products. At present, most studies of consumer embarrassment and embarrassing products start from consequences and coping methods. Most studies have shown that if consumers feel “embarrassing” it has a negative impact on subsequent purchase willingness and behavior (Penz and Stoettinger, 2004). If an individual encounters an embarrassing event, individuals will think that embarrassing events will have a negative impact on their social image, and individuals tend to take steps to enhance self-image (Feinberg et al., 2012); however, few studies have focused on the embarrassing product itself. This study fills this gap by studying the image type of embarrassing products, starting from the product packaging element design, discussing which image type to use is the most likely to reduce the embarrassing perception of the embarrassing product itself and promote consumers’ willingness to buy embarrassing products.

Finally, this research contributes to the literature on product packaging design. At present, studies have investigated that many elements of product packaging can affect consumer perception (such as image, color, weight, shape, etc.). For images, previous studies focused on location, brightness, contrast, and color (Gil-Perez et al., 2020), but few studies on image types (especially natural/artificial images). Some studies suggest that expectations can be enhanced when natural images are used (Kim et al., 2019), but other studies suggest the opposite effect, that artificial images can enhance expectations for certain product attributes (Cai and Chi, 2021). The opposite effect is likely to be the absence of product categories. Therefore, this study focuses on the product packaging image types of embarrassing product categories to provide empirical evidence for related research on image types.

### Practical Implication

This study also provides some enlightenment for the practice of embarrassing products marketing. On the one hand, the current research provides a reference for businesses’ packaging design of embarrassing products. While most studies have shown that eye-catching packaging is a good way to increase sales, the results of this study suggest that a product packaging design that attracts the attention of consumers may have negative effects for embarrassing products. When making product packaging image design, businesses should try to choose artificial images, such as illustrations and cartoons, to reduce the awakening of consumers to negative body imagery and promote the sales volume of embarrassing products.

On the other hand, this study also provides support for the design of embarrassing product advertising language. If businesses use natural images when designing packaging for embarrassing products, they are best to choose third-person descriptions when designing the advertisement. Because the first-person instruction pronouns will make consumers think that information is for consumers themselves, more likely to associate with their defective body, feel more negative body imagery. But in the third-person pronouns, the likelihood of consumers arousing the negative body imagery will be greatly reduced, thus effectively reducing the negative impact of images on the willingness to buy.

### Limitation and Future Research Direction

There are still some limitations to this research, which can be further improved in future research. On the one hand, the factors that influence consumers to buy embarrassing products are complex, and some of them may influence the results are not taken into account. For example, from the perspective of the individual, consumers’ values, self-construction, and other personal factors may be added into this study as moderator variables. Some external factors, such as advertising framework, the level of specificity of advertising (vs. abstraction), and even the social interaction in the consumer scene may also play a moderating role, which could be explored in future research. On the other hand, although embarrassing products are generally highly related to the body field, some product categories involve personal privacy, so the images on these product packaging generally do not have images of body parts, such as condoms, impotence drugs, etc. The conclusions drawn in this study may not apply to the packaging design of these embarrassing products, but relevant studies on them will also be inspired. Through the exploration of this phenomenon, it can be found that advertisements for such embarrassing products often use anthropomorphic means. For example, the famous condom producer “Jissbon” has designed its logo image to be similar to a person with sunglasses. Similarly, “Durex” conveys hidden content and product information through the virtual image of “DuDu” in a lively and relaxed tone on online social media platforms. Anthropomorphic designs can not only improve product evaluation and attitude but also enhance product attractiveness (Laksmidewi et al., 2017). Combined with the conclusions of this study, the artificial perception of images can improve embarrassing products’ purchase intention. Therefore, we speculate that the use of anthropomorphism in advertisements can weaken the embarrassment of consumers when they watch the advertisements of embarrassing products. However, some studies have pointed out that anthropomorphism will also bring negative effects (Hur et al., 2015). For example, inappropriate anthropomorphic design of food objects in advertisements will make people feel guilty (Di and Haizhong, 2017). Therefore, future research can further explore this proposition.

## Data Availability Statement

The raw data supporting the conclusions of this article will be made available by the authors, without undue reservation.

## Ethics Statement

Ethical review and approval was not required for the study on human participants in accordance with the local legislation and institutional requirements. The patients/participants provided their written informed consent to participate in this study. Written informed consent was obtained from the individual(s) for the publication of any potentially identifiable images or data included in this article.

## Author Contributions

SY conceived and designed the experiments. SY and HY carried out the experiments and analyzed the experimental results. ZL wrote the manuscript. HY edited the manuscript. ZH provided constructive suggestions for the revision of the manuscript.

## Conflict of Interest

The authors declare that the research was conducted in the absence of any commercial or financial relationships that could be construed as a potential conflict of interest.

## Publisher’s Note

All claims expressed in this article are solely those of the authors and do not necessarily represent those of their affiliated organizations, or those of the publisher, the editors and the reviewers. Any product that may be evaluated in this article, or claim that may be made by its manufacturer, is not guaranteed or endorsed by the publisher.

## Footnotes

1
https://npcitem.jd.hk/100020137176.html

4
https://www.hellorf.com/image/show/hi2240393380?source=search_file

5
https://www.1688e.com/project/liangjianyaodian/youshi/

6
https://npcitem.jd.hk/10037173988946.html?gjz=0

7
https://static.amazon.cn/dp/B07PM7SVDP/ref=sr_1_54?keywords=YNSADIET &qid=1641437788&sr=8-54

9
https://www.loandd.com/huangqi/gongxiao/1711.html
